# Physiological and physical characteristics of BMX freestyle athletes: a preliminary review

**DOI:** 10.3389/fphys.2025.1633217

**Published:** 2025-08-21

**Authors:** Weibao Liang, Yu Hou, Chuannan Liu, Shuhui Ma, Yue Zong, Xujie Yan

**Affiliations:** ^1^ School of Physical Education and Sports Science, Hengyang Normal University, Hengyang, China; ^2^ School of Physical Education and Sports Science, South China Normal University, Guangzhou, China; ^3^ Department of Physical Education, Kunsan National University, Gunsan-si, Republic of Korea; ^4^ Zunyi Mdedical University, School of Physical Education and Health, Zunyi, China; ^5^ School of Physical Education, Guangdong Technology College, Zhaoqing, China

**Keywords:** BMX freestyle, physiological characteristics, physical characteristics, strength and conditioning, action sports, performance

## Abstract

BMX Freestyle, a newly recognized Olympic discipline, demands athletes perform intricate, high-intensity maneuvers during 60-s competitive runs. Despite the sport’s rapid evolution, there is a notable scarcity of scientific investigation into the distinct physiological and physical attributes of its athletes. This preliminary review synthesizes the extant literature to delineate the key physiological and physical characteristics of BMX Freestyle athletes and to identify pressing directions for future research. Elite male athletes typically present with lower body fat percentages, contributing to an advantageous power-to-weight ratio. Physiologically, these athletes demonstrate substantial anaerobic power, crucial for executing aerial maneuvers and complex rotational skills. Maximal oxygen uptake (VO_2_max) is typically moderate, likely facilitating inter-competition recovery and the capacity to sustain demanding training regimens. Musculoskeletally, athletes require high lower-limb explosive power for jump amplitude, significant upper-body strength for bike control and landing impact attenuation, and robust core musculature for executing complex aerial rotations. Furthermore, highly developed neuromuscular control, including dynamic balance, precise muscle activation patterns, and coordination, is foundational for performing advanced skills. This synthesis provides an evidence-based framework for optimizing training protocols focused on explosive power and eccentric strength, developing quantitative talent identification models, and implementing targeted injury prevention strategies that address the unique demands of the human-bicycle interface. Future research should focus on validating these applications and investigating the characteristics of female competitors to advance athlete health and performance in this evolving Olympic sport.

## Introduction

BMX Freestyle is distinguished as an expressive athletic endeavour wherein participants execute sequences of diverse manoeuvres, constituting a “routine,” across a range of purpose-built terrains. Under the governance and promotional efforts of the Union Cycliste Internationale (UCI), its Park discipline has achieved formal inclusion as an Olympic event. The venue for Park competitions is typically configured as a square or rectangular area, incorporating a variety of obstacles such as Quarter Pipes, Spine Ramps, Jump Boxes, Hip Jumps, and Rails. A requisite feature of these courses is the inclusion of at least three central obstacles, complemented by further impediments distributed around the perimeter of the performance area. Within this structured environment, athletes undertake 60-s “runs” to demonstrate an array of technical skills, which may include Spins, Flips, Bar spins, Tailwhips, and Wheelies, among others; Table 1 provides a more comprehensive overview of common obstacles encountered in these park environments. Adjudicators evaluate the athletes’ overall performance based on a multi-dimensional framework, encompassing criteria such as difficulty, aerial elevation, fluidity, originality, execution consistency, strategic utilisation of the course, undertaken risk, and the quantitative repertoire of tricks, with scores allocated on a scale from 0.00 to 99.99 (Olympics, 2024). The bicycles employed in Park competitions are relatively compact and lightweight, customarily equipped with 20-inch (approximately 50 cm) wheels. Frames are constructed from materials optimized for low mass, such as aluminium alloys, enabling the complete bicycle to weigh as little as 12 kg. This design paradigm is intentionally geared towards enhancing rider manoeuvrability during the execution of complex technical skills (Olympics, 2017).

**TABLE 1:** Some of the common obstacles that can be found on these courses.

Basic tricks	Description
Quarter Pipe	This ramp is shaped like one-fourth of a round pipe. These may be placed against a higher wall along the edges of the course, banking up to a wall, or there may be a flat surface at the top of the quarter pipe. On beginner courses, these ramps are 1.2–1.8 m (4–6 feet) tall.
Spine Ramp	This is made by putting two-quarter pipes back to back, creating a narrow spike—or “spine”—where the two ramps meet.
Jump Box	Unlike a spine ramp, this box has uneven sides—the “jump” side is steeper than the landing side, making it easier for a rider to land on the other side and continue riding smoothly.
Hip Jump	This obstacle is formed by linking two-quarter pipes at an angle.
Rails	Metal rails may line the ramps or exist on their own, like a stair rail or a rail that’s perpendicular to the ground. These rails can be used by riders to grind, or slide, across or down. They may also ride on the rail with either their front or back wheel.

BMX Freestyle is distinguished by its extreme acrobatic complexity and dynamism. Athletes are required to execute difficult aerial manoeuvres, including rotations, flips, and whips, whilst navigating diverse obstacles at high velocity through jumps and glides. The sport is described as “energetic” and “exhilarating and fast-paced.” Successful execution of these maneuvers places multifaceted and extreme demands on the athlete’s physiological systems and neuromuscular control (Albaladejo-García et al., 2023). Due to the absence of shock-absorption systems on BMX bicycles, athletes must also rely on precise body control for landings, rendering postural accuracy paramount. These demanding characteristics underscore the need for a detailed scientific examination of the specific attributes required for elite performance.

BMX Freestyle originated from BMX racing, where racers initially performed tricks during competitive interludes, ostensibly for leisure (Edwards and Ugo Corte, 2009). Freestyle BMX exhibits parallels with mountain biking, demonstrating suitability for managing loose substrates and executing jumps (Olsen, 2021). Distinct from traditional BMX racing, which prioritizes speed and sprinting capabilities, the core competitive value of BMX Freestyle resides in the comprehensive and innovative execution of tricks. Over time, the sport has evolved and achieved widespread recognition; its incorporation into major events such as the X-Games and the Olympic Games has further solidified its legitimacy and professional standing. Nevertheless, dedicated research concerning the physiological and physical characteristics of BMX Freestyle athletes, particularly those competing in its Park discipline, remains relatively scarce. A literature review encompassing 87 BMX-related publications from 1982 to 2022 indicated that most studies were concentrated in biomechanics and physiology/sport science, primarily addressing performance enhancement in BMX racing, with merely 18 focusing on freestyle disciplines (Camilleri et al., 2024).

This current research landscape underscores the imperative to consolidate existing data and delineate future research trajectories. Although historical connections to BMX racing may provide some transferable insights, the unique movement patterns and skill requisites inherent to freestyle disciplines dictate specific athletic demands that necessitate focused investigation. Therefore, the primary objective of this review is to synthesize and critically evaluate the current scientific literature to establish a comprehensive profile of the key physiological and physical characteristics of elite BMX Freestyle athletes. To achieve this, we will examine a range of pivotal attributes—from anthropometric profiles and energy system contributions to the specific musculoskeletal and neuromuscular demands of the sport—in order to build a foundational evidence base and identify critical directions for future applied research.

## Methods

This preliminary review synthesized existing scientific and official literature to delineate the physiological and physical characteristics of BMX Freestyle athletes. A primary search was conducted in major academic databases, including PubMed, Scopus, Sportdiscus and Cochrane Library, for literature published up to May 2025. The search strategy employed a combination of keywords specific to the core topic, such as “BMX Freestyle,” “freestyle cycling,” “physiology,” “biomechanics,” “physical characteristics,” “strength,” “power,” and “aerobic capacity.” To ensure a comprehensive understanding of the sport’s regulations, competitive format, and trick definitions, this database search was supplemented with information from the official websites of the International Olympic Committee (IOC) and the Union Cycliste Internationale (UCI).

### Inclusion and exclusion criteria

Studies and official documents were included if they provided data or descriptions related to the physiological, physical, anthropometric, or biomechanical attributes of BMX Freestyle athletes. Due to the limited body of literature specific to BMX Freestyle, peer-reviewed studies on BMX Racing were consulted for comparative purposes where relevant (e.g., for foundational physiological benchmarks), but the primary focus of this review remains exclusively on the Freestyle discipline. For the comparative analysis section, targeted secondary searches were conducted to identify literature on sports with similar physical demands. Keywords for these searches included “artistic Gymnastics,” “skateboarding,” “trampoline,” and “freestyle skiing.” Studies were excluded if they focused solely on injury epidemiology without performance data, were non-academic publications (excluding the aforementioned official governing body websites).

### Data extraction and synthesis

Key quantitative data points were extracted, including but not limited to, anthropometric measurements (height, weight, body fat %), anaerobic power (e.g., Wingate test results), maximal oxygen uptake (VO_2_max), and muscular strength (e.g., vertical jump). Qualitative information regarding technical execution, competitive structure, and specific skill demands was also systematically gathered. The findings were then thematically synthesized and organized according to a conceptual framework that categorizes athletic attributes into: 1 Physical Characteristics, 2 Physiological Characteristics (encompassing energy systems, musculoskeletal traits, and neuromuscular control), and 3 Biomechanical and Technical Considerations. Data from analogous sports were extracted and integrated into the “Comparative Perspectives” section to contextualize the findings and highlight the unique demands of BMX Freestyle.

## Physical characteristics

Athletic performance in dynamic disciplines such as BMX Freestyle is substantially influenced by athlete anthropometry and body composition. Research on the physical characteristics of BMX Freestyle athletes is presently limited, though available data offer a preliminary profile. A study of seven male professional BMX Freestyle athletes (Moreland, 2010) yielded valuable data: mean age 25.85 ± 1.86 years, height 173.44 ± 6.20 cm, and body mass 76.51 ± 8.53 kg, with a mean body fat percentage of 15.32% ± 4.27% determined via a 3-site skinfold assessment.

In the context of Park competition, the power-to-weight ratio is particularly emphasized, as excess body mass can detrimentally affect aerial height and manoeuvrability during complex tricks. A lean physique aids in reducing inertial load during rapid directional changes and aerial rotations, thereby enhancing overall efficiency in dynamic movements. While this body fat level is not exceedingly low, in conjunction with their body mass, it suggests athletes maintain strength while possessing adequate mass to manage landing impacts and sustain aerial stability.

Although substantial research has focused on BMX racing athletes, many findings can be extrapolated to the unique demands of Park competition. Studies on BMX athletes commonly report lean, muscular physiques with a high muscle-to-fat ratio (Mateo et al., 2011; Pavlović et al., 2022). Another investigation (Albaladejo-García et al., 2023) involving 19 international-level BMX athletes (seven freestyle, 12 racing; seven female) reported overall mean values of: age 21.9 ± 4.4 years, height 170.6 ± 9.9 cm, and body mass 68.6 ± 12.1 kg. It is noteworthy that these data combine athletes from disparate disciplines and sexes, thereby possessing limited specific representativeness for freestyle athletes.

## Physiological characteristics

BMX Freestyle, an action sport demanding a confluence of technical mastery, explosive power, and creative expression, places distinctive and exacting physiological demands upon its practitioners. Such requirements span multifaceted physiological domains, encompassing energy metabolism, muscular performance characteristics, and intricate neuromuscular regulation.

### Energy system contributions

BMX Freestyle competition runs are of short duration, typically 60 s, during which athletes execute a series of high-intensity, explosive manoeuvres. This characteristic indicates a primary reliance on the anaerobic metabolic system for energy provision. Data from a study on male professional BMX Freestyle athletes demonstrated substantial anaerobic power, with a 30-s Wingate ergometer test revealing a mean peak power of 802.50 ± 91.44 W and a relative peak power of 10.49 ± 1.03 W/kg (Moreland, 2010). For comparison, elite BMX racing athletes also exhibit notable anaerobic capabilities, with short-distance sprint peak power reaching 1,498 ± 189 W and Wingate mean power at 1,344 ± 158 W (Petruolo et al., 2020). Although specific sporting demands differ between racing (e.g., explosive gate starts, multiple track sprints) and freestyle, high power output capability is a core commonality in BMX disciplines reliant on explosive performance. The high power values exhibited by freestyle athletes in Wingate tests directly corroborate the critical importance of anaerobic power for achieving air, maintaining aerial posture, and executing complex manoeuvres. The power-to-weight ratio (W/kg) is particularly crucial in sports involving gravity defiance for aerial actions.

The same cohort of male professional BMX Freestyle athletes recorded a maximal oxygen uptake (VO_2_max) of 43.48 ± 6.10 mL/kg/min (Moreland, 2010). This value is modest compared to elite endurance athletes and lower than that of elite BMX racers (VO2max approximately 55.7 ± 4.8 mL/kg/min) (Petruolo et al., 2020). While a single 60-s run is predominantly anaerobic, competitions typically involve multiple rounds (e.g., qualifications, finals) with 8–15 min rest intervals. A VO2max of approximately 43 mL/kg/min, though not indicative of elite endurance capacity, remains important for supporting repeated high-intensity efforts. A well-developed aerobic system facilitates phosphocreatine resynthesis and the clearance of metabolic byproducts (e.g., lactate, though lactate levels in freestyle athletes are not directly reported in the literature), thereby enabling athletes to maintain high performance quality in subsequent runs. Consequently, even though aerobic capacity is not the primary determinant of performance, aerobic endurance development should not be entirely disregarded in training. Furthermore, mastering and refining tricks necessitates extensive repetitive practice by athletes. These practice sessions are often of extended duration, involving numerous attempts, ramp ascents, and short, intense bursts of activity. This cumulative training load imposes demands on the aerobic system to sustain training volume, delay fatigue, and maintain practice quality. This implies that training periodization should modulate the emphasis on different energy systems according to the proximity of competition.

### Musculoskeletal characteristics

The strength characteristics of BMX Freestyle athletes exhibit highly specialized demands, with an emphasis on the optimization of functional strength and power-to-weight ratio, rather than the mere pursuit of maximal absolute strength. Athletes utilize a “pumping” motion, a squat-like mechanism performed on ramp transitions, to generate speed, momentum, and ultimately, aerial height; this maneuver places considerable demands on both lower-limb and core musculature strength. Concurrently, upper-body strength is instrumental in maintaining balance, stability, and vehicular control.

#### Lower-limb explosive power

Central to BMX Freestyle performance is the athlete’s capacity for rapid force production and application. The requisite lower-limb explosive power is critical for generating the impulse necessary for successful ramp take-offs and for producing vertical lift during aerial maneuvers. A mean vertical jump height of 54.25 ± 4.42 cm reported for male freestyle athletes (Moreland, 2010) substantiates their proficient lower-limb explosive capabilities. Wingate peak power output, previously addressed in the context of anaerobic capacity, also serves as a pertinent indicator of lower-limb explosiveness. The inherent nature of the sport, which involves athletes maneuvering over obstacles such as ramps, jumps, and rails, necessitates exceptionally high power outputs. Explosive power, particularly the coordinated application of force from the lower limbs in conjunction with upper-body actions, underpins the amplitude of maneuvers, a key component in judging criteria.

#### Upper-body, grip, and core stability

Muscular strength is considered “paramount” in BMX Freestyle (Oller, 2012), as it underpins powerful pedaling, execution of high-difficulty jumps, and the maintenance of body control and balance during skill performance, engaging leg, core, and upper-body musculature. Superior upper-body strength is essential for handlebar manipulation, the execution of aerial maneuvers such as barspins and tailwhips, and for dynamic control and stability during landing impact absorption. Specifically, during “no-footed” tricks, robust and sustained grip strength is critical for maintaining vehicular control and ensuring rider safety. Notably, when compared to elite athletes in sports such as rugby, baseball, and basketball, BMX riders have exhibited relatively lower upper-body strength, suggesting a potential area for targeted development (Moreland, 2010). This observation, however, may indicate that BMX Freestyle prioritizes functional strength—emphasizing power-to-weight ratio and dynamic control—over the maximal absolute strength commonly observed in some field and court sports. Excessive muscular hypertrophy, if not translated into effective on-bike performance, could potentially compromise agility due to increased body mass. Although direct investigations employing “core strength tests” in BMX Freestyle athletes are lacking, the necessity to perform complex aerial rotations and maintain stable vehicular control, coupled with electromyography (EMG) findings demonstrating high rectus abdominis activation during flips and rotations, underscores the foundational role of a potent core. Research (Voronov et al., 2020) indicated that with increasing trick difficulty, particularly maneuvers involving body rotation, the myoelectrical cost to the rectus abdominis rises significantly, which aids in torso stabilization and reduction of the moment of inertia. This implies that a robust and stable core is indispensable for initiating, modulating, and arresting complex rotational movements in mid-air.

#### Muscular endurance

Notwithstanding the relatively brief duration of a single competitive run (60 s), the imperative to execute a sustained series of high-energy maneuvers with consistent quality within this timeframe, and to maintain high performance levels across multiple runs in both competition and training, places considerable demands on muscular endurance. Specifically, the local muscular endurance of key muscle groups—such as the quadriceps, core musculature, and upper-body muscles—is critical for resisting fatigue, preserving movement precision, and ensuring stable vehicular control.

### Neuromuscular control and skill execution

Superior performance in BMX Freestyle is critically contingent upon a highly refined neuromuscular control system. This system encompasses balance proficiency, the precise modulation of muscle activation patterns, and rapid adaptive and reactive capabilities.

#### Balance: static vs. dynamic control

A study (Albaladejo-García et al., 2023) involving 19 international BMX athletes (7 freestyle, 12 racing) and 20 physically active adults, which utilized a single-leg stance balance test, revealed that BMX athletes did not demonstrate superior performance compared to the control group concerning static balance parameters such as center of pressure (COP) dispersion and velocity. Indeed, under specific conditions (e.g., when the leading leg was considered dominant), the athletes exhibited greater mediolateral COP variability. Conversely, and in contrast to the control group, BMX athletes displayed no significant balance asymmetry between their dominant and non-dominant legs. This somewhat counterintuitive finding suggests that conventional static single-leg balance assessments may inadequately capture and evaluate the highly dynamic, task-specific balance capabilities requisite for BMX Freestyle. Nevertheless, the manifestation of balance asymmetry in cycling contexts remains poorly understood. In contrast, rhythmic gymnasts have demonstrated asymmetry (Shigaki et al., 2013), potentially because they consistently utilize the same supporting limb for various technical elements, including balances and turns. In the aforementioned study (Albaladejo-García et al., 2023), the control group exhibited asymmetry during single-leg stance, with the dominant leg showing significantly poorer balance performance than the non-dominant leg. Contradictory findings have also been reported in studies related to sports such as soccer (King and Wang, 2017). Soccer players typically favor their more proficient (dominant) leg for kicking, while their non-dominant leg assumes a stabilizing role to enhance accuracy during execution. Consequently, the non-dominant leg often demonstrates superior balance parameters due to its high degree of specialization in these repetitive actions. Although BMX riders consistently position the same leg forward to stabilize the bicycle during jumps and tricks, this did not translate into significant balance asymmetries in the study (Albaladejo-García et al., 2023). It is plausible that this cohort may exhibit asymmetries in other skills, such as unilateral jumping or force application (Maloney, 2018). Significantly, no notable differences were identified between the racing and freestyle BMX modalities across any of the investigated balance metrics in that study.

The “balance” exhibited by athletes during BMX Freestyle competition is likely a more comprehensive capability, involving the complex integration and dynamic interplay of proprioceptive, vestibular, and visual inputs, alongside rapid muscular co-adjustments. This intricate process unfolds while maneuvering the bicycle at high speeds through complex obstacle courses. This underscores the critical need for future research to develop balance assessment methodologies with greater ecological validity, capable of simulating authentic sport-specific scenarios.

#### Muscle activation patterns

Research by Voronov et al. (2020) utilized electromyography (EMG) to investigate muscle activation patterns during the execution of BMX Freestyle maneuvers. Key findings from this investigation indicated that: 1 With escalating trick difficulty - encompassing a wide repertoire of technical elements such as those detailed in Table 2, there was a significant augmentation in EMG activity of upper limb flexors (e.g., brachioradialis, biceps brachii) and core musculature (e.g., rectus abdominis). 2 Rectus abdominis activity was particularly pivotal during the execution of flipping maneuvers and techniques involving handlebar or bicycle frame rotations; its heightened activation is critical for torso stabilization, effective power transmission, and precise control of body rotation. 3 Concomitant with increased rectus abdominis activation, there was a relative reduction in the myoelectrical activity of its antagonist muscle groups (e.g., trapezius, latissimus dorsi), reflecting an efficient pattern of synergistic and antagonistic muscle co-activation. 4 The EMG-derived muscular cost for the vastus lateralis and biceps femoris (key leg muscles) was more substantially influenced by jump height (i.e., the demands of landing impact absorption) than by the intrinsic complexity of the trick itself. These EMG analyses provide invaluable insights into the specific muscular demands of various tricks and how muscle recruitment strategies adapt to changing task requirements. Such information holds considerable applied value for designing targeted strength and conditioning protocols aimed at reinforcing prime movers and stabilizing muscles crucial for key actions. Furthermore, these findings can inform injury prevention and rehabilitation strategies; for instance, given the critical role of the rectus abdominis in flips, ensuring its adequate strength and endurance, along with coordinated relaxation of antagonist muscles, may mitigate the risk of compensatory movements or excessive loading on other anatomical structures.

**TABLE 2:** Technical elements in freestyle BMX.

Basic tricks	Description
Spins	Riders may perform a 360 spin, spinning the whole way around, or performing multiple spins-a 720, which is two spins, or 1,080, which is three.
Bunny hop	The rider jumps the bike into the air to gain maximum height.
Flips	Riders do much more than single backflips: They can perform double backflips, and even front flips.
Barspins	While performing another flying element, a rider may spin their handlebars around once, or multiple times.
Tailwhips	In a tailwhip, the rider holds the handlebars while removing their feet from the pedals spinning the frame of the bike out from under them, eventually winding up with it back under their butt and legs. Some tricks involve multiple tailwhips.
Wheelies	Athletes may ride on the front or the back wheel, with the other wheel in the air.

#### Agility, coordination, and reaction time

Although the extant literature lacks specific data from standardized assessments of agility, coordination, and reaction time in BMX Freestyle athletes, these attributes are unequivocally central to the sport’s demands. The execution of intricate trick sequences within minimal timeframes, adaptable navigation of diverse course obstacles, and the necessity for precise, instantaneous adjustments during high-speed motion collectively impose extreme demands on an athlete’s coordination, agility, and rapid reactive capabilities. The Functional Movement Screen (FMS) has been cited as a potential tool for evaluating movement patterns, stability, flexibility, strength, and coordination, as well as for detecting asymmetries (Sulowska-Daszyk et al., 2015); however, its application within the BMX Freestyle athlete population has not been documented. The unpredictable nature of dynamically navigating a course and linking tricks underscores the heightened requirement for these neuromuscular qualities. The absence of pertinent test data in this domain represents a significant research lacuna.

#### Neuromuscular fatigue

The confluence of high power outputs, intricate coordination, and high-intensity muscular activations during 60-s competitive runs, particularly when repeated across multiple rounds, is highly conducive to the onset of neuromuscular fatigue. This fatigue can subsequently impair the quality of skill execution and elevate the risk of injury. Although not directly quantified in existing studies, the documented physiological demands (Moreland, 2010) and observed muscle activation patterns (Voronov et al., 2020) strongly suggest a substantial neuromuscular load. As fatigue ensues, an athlete’s capacity for precise motor control, rapid reaction, and balance maintenance is likely to deteriorate. This factor is of particular concern given the inherently high-risk nature of BMX Freestyle.

## Biomechanical and technical considerations

### Integration of physical attributes and technical skill

Performance in BMX Freestyle is contingent not merely on inherent physiological or physical attributes, but critically on the athlete’s capacity to integrate these traits with refined technical skills. The execution of complex aerial maneuvers, such as tailwhips, barspins, and flips, necessitates precise timing, intricate bodily coordination, and a sophisticated understanding of bicycle-rider dynamics (Scott, 2013). Technical proficiency is frequently underpinned by the athlete’s physiological preparedness, whereby optimal force generation—governed by the force-velocity relationship—is translated into the explosive upper and lower limb movements required for trick execution. The effective transmission of muscular force, as evidenced by research correlating vertical jump performance with BMX-specific cycling power output, underscores the interdependence between physical conditioning and technical execution (Gross and Gross, 2019). Furthermore, video-based kinematic analyses suggest that pedaling cadence and coordination, aerial bike handling during jumps, and the ability to modulate power output during transitions are critical determinants of competitive success in BMX (Cowell et al., 2012; Mateo-March et al., 2012).

### Equipment and its influence on performance

The physical and physiological demands of BMX Freestyle Park are inextricably linked to the equipment utilized by athletes. BMX Freestyle Park bicycles are typically configured with specific modifications, such as lowered seat posts (often ‘slammed’ to enhance maneuverability) and the inclusion of a gyroscopic detangler system to manage brake cable entanglement, thereby augmenting rider responsiveness and control during technical maneuvers (Scott, 2013). Such equipment adaptations enable riders to maximize power output while preserving the fine motor control requisite for executing complex actions. The interplay between the athlete and their equipment also dictates how physiological capabilities, such as neuromuscular efficiency and explosive power, are expressed during performance. These considerations underscore the necessity for integrated training methodologies that address both physiological conditioning and technical proficiency in relation to equipment usage.

## Comparative perspectives

While the corpus of research specifically targeting BMX Freestyle remains circumscribed, valuable insights can be gleaned from studies in analogous sporting disciplines. For instance, investigations into the physical fitness characteristics of elite freestyle skiing aerialists have underscored the significance of lower-limb explosive power, core stability, and an exceptional power-to-weight ratio (Yao and Niu, 2024). Although scoping reviews (Camilleri et al., 2024) have synthesized research trends in BMX, they highlight a discernible gap in targeted studies addressing the nuanced physiological and physical attributes specific to BMX Freestyle athletes.

To provide a more structured and detailed comparison, the physiological and physical characteristics of BMX Freestyle athletes are contextualized alongside several analogous action sports in Table 3. This table synthesizes available quantitative data with qualitative descriptions of key performance demands. It is important to note that the very act of constructing this comparison highlights a significant finding: while traditional disciplines like artistic gymnastics have established physiological profiles, emerging Olympic sports such as skateboarding and even trampoline have a notable scarcity of comprehensive, publicly available data, a gap that mirrors the current state of BMX Freestyle research.

**TABLE 3:** Comparative profile of physiological and physical characteristics across action sports.

Parameter	BMX Freestyle (Moreland, 2010)	Skateboarding (Park) (Ab Rasid et al., 2024)	Freestyle Skiing (Aerials) (Yao and Niu, 2024)	Artistic Gymnastics (Ghosh et al., 2025; Heller et al., 1998)	Trampoline (Dyas et al., 2023; Sun et al., 2024)
Body Fat %	15.3% (Male)	Low body fat is advantageous, though normative data is scarce.	Male: 10.9% Female: 19.3%	Male: ∼9–11% Female: ∼13–16%	Low body fat is critical for performance, but specific mean data is limited.
VO_2_max (mL/kg/min)	43.5 (Male)	Moderate aerobic fitness is likely required for recovery.	52.8 (Male)	Male: ∼45–53 Female: ∼40–42	Aerobic fitness positively correlates with performance, but mean data is scarce.
Vertical Jump (cm)	54.3 (Male)	95.5 (Male)	Male: 58.7 Female: 44.9	∼45–55 (Male and Female)	∼41–42 (Elite)
Balance (Static and Dynamic)	Crucial	Paramount	Exceptional	Paramount	Paramount
Lower-Limb Strength	High	High. Leg strength is an important performance predictor.	Very High	Very High	Maximal force and explosive strength are key determinants of performance
Core Strength	Crucial	Paramount	Exceptional	Paramount	Paramount
Power-to-Weight Ratio	Advantageous	High	Exceptional	Paramount	Very High
Primary Energy System	Anaerobic	Anaerobic	Anaerobic	Anaerobic	Anaerobic

“Paramount” indicates a characteristic that is arguably the most fundamental requirement for the sport. This table aims to provide a conceptual comparison using the best available data to highlight the unique profile of the BMX, freestyle athlete.

**FIGURE 1:**
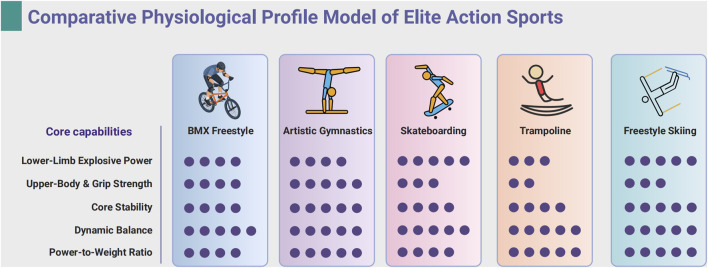
Comparative physiological profile model of elite action sports. Comparative Physiological Profile Model of Elite Action Sports. The profiles are based on a 1-5 qualitative scale derived from the quantitative and descriptive data synthesized in Table 3. The scale is defined as follows: 1-Low, 2=Moderate, 3-High, 4-Very High, 5-Paramount/Exceptiona. Each dot represents a score point on a 1-5 scale. Each sport's rating on the five core capabilities (Lower-Limb Explosive Power, Upper-Body and Grip Strength, Core Stability, Dynamic Balance, Power-to-Weight Ratio) was assigned based on a synthesis of the available literature to allow for a conceptual visual comparison of their athletic signatures. Created in BioRender.

As illustrated in Table 3, a common athletic profile emerges across these disciplines, characterized by a dominant anaerobic energy system, a high power-to-weight ratio, and highly developed neuromuscular control. However, the table also illuminates subtle but critical distinctions. For instance, while all sports require exceptional core strength and explosive power, artistic gymnastics may demand even lower body fat percentages and greater static strength for apparatus-based elements. Conversely, BMX Freestyle presents a unique challenge in its human-bicycle interface, requiring significant upper-body and grip strength to manipulate the vehicle during aerial maneuvers and absorb landing impacts—a demand less pronounced in bodyweight-only sports like gymnastics and trampoline. To further visualize the distinct athletic signatures outlined in Table 3, a comparative profile model is presented in Figure 1. This figure graphically represents each sport’s strengths across five core capabilities, allowing for an immediate conceptual comparison of their unique physiological profiles.

To understand the biomechanical basis for these distinctions, it is useful to deconstruct how key skills are performed in each discipline:

### Generation and application of explosive power

The generation of explosive power for aerial maneuvers is a common requirement, yet the biomechanical strategies differ significantly.

• In artistic gymnastics, athletes generate vertical impulse from a stable, predictable surface (e.g., a sprung floor), propelling only their body mass.
• In skateboarding, the explosive “pop” is a distinct mechanism, propelling the athlete plus a very light board (approx. 2–3 kg).
• In BMX Freestyle, athletes must propel a combined human-bicycle system (upwards of 80–90 kg). Power is generated through a whole-body effort involving both lower-limb “pumping” and significant upper-body pulling on the handlebars.

### The nature of balance and neuromuscular control

Balance is paramount in all disciplines, but its nature and the control strategies employed are fundamentally different.

• Gymnastics demands control of the body’s center of mass relative to a stable base of support.
• Skateboarding requires constant corrections on an inherently unstable, rolling surface, a task dominated by the lower limbs.
• BMX Freestyle presents a compound balance challenge within a complex human-bicycle dynamic system, controlled through four points of contact (hands and feet), where upper-body and grip strength are critical for micro-adjustments.

## Discussion

**FIGURE 2:**
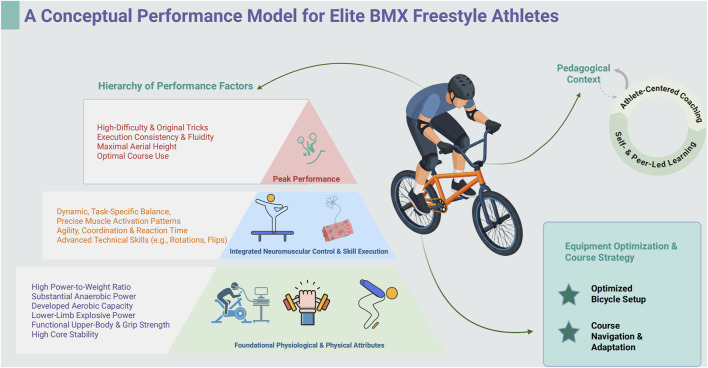
A conceptual performance model for elite BMX freestyle athletes. This model illustrates the hierarchical and interactive factors contributing to elite performance. The central pyramid depicts the core athletic competencies, where foundational physiological and physical attributes (base layer) support the integrated neuromuscular control and execution of technical skills (middle layer), which culminate in peak performance outcomes (top layer). These core competencies are critically influenced by two external contextual factors: the unique, athlete-centered Pedagogical Context in which skills are acquired, and the Equipment Optimization and Course Strategy which dictates how physical abilities are expressed. Created in BioRender.

The synthesis of literature in this review culminates in a holistic understanding of the multifaceted demands placed upon the elite BMX Freestyle athlete. To visually represent the interplay of these demands, a conceptual performance model is presented in Figure 2. This model provides a hierarchical framework, illustrating how foundational physical attributes are integrated through neuromuscular control and influenced by external factors to produce elite-level performance. The following discussion will deconstruct the key components of this model, beginning with a synthesis of the core athlete profile.

### Synthesis of the elite BMX freestyle athlete profile

Elite BMX Freestyle athletes reportedly require a multifaceted physiological profile, encompassing high levels of flexibility, explosive power, strength, and both anaerobic and aerobic capacities, to underpin their competitive performance (Oller, 2012). These physiological attributes are typically intrinsically linked to the athlete’s specific somatotype. Broadly, BMX Freestyle imposes distinct physical demands on its participants: 1 A reduced body fat percentage and a lean physique are conducive to an enhanced power-to-weight ratio, which is paramount for executing high-difficulty aerial maneuvers, achieving greater jump amplitude, and augmenting overall agility (Pavlović et al., 2022). 2 Athletes require substantial anaerobic capacity, a muscle fiber composition predominantly favoring fast-twitch characteristics for rapid force generation, and an optimized force-velocity profile to enable powerful take-offs and effective execution of aerial skills (Gross and Gross, 2019). 3 Equally vital are physical attributes such as robust core strength, superior dynamic balance, and pronounced agility, which empower riders to execute complex stunts and transition seamlessly between them (Bach et al., 2015; Yao and Niu, 2024). The synergy between these physiological and physical characteristics, augmented by advanced technical proficiency and efficacious equipment utilization, forms the cornerstone of high achievement in BMX Freestyle. Elite athletes are tasked with integrating a diverse array of physical capacities within exceedingly narrow time windows: these include explosive power for attaining sufficient aerial height, precise vehicular control, exceptional balance, robust core stability (particularly crucial for executing complex rotational maneuvers), and artistically expressive choreography of their routines. Notwithstanding the “freestyle” designation of the sport, high-level competitive performances typically involve meticulously choreographed routines, requiring athletes to flexibly adapt and execute predetermined sequences with high fidelity under variable course conditions. This dialectical interplay between improvisation and pre-planning imposes substantial demands on an athlete’s cognitive processing capabilities, real-time decision-making skills, and capacity for execution under pressure.

### The unique athletic signature of the BMX freestyle athlete

The findings of this review confirm that elite BMX Freestyle athletes share a common foundation with other high-intensity acrobatic sports, necessitating high power-to-weight ratios, dominant anaerobic energy systems, and exceptional neuromuscular control. However, a more critical comparison reveals that the physiological and physical demands of BMX Freestyle are not merely a variation of other disciplines but constitute a unique athletic signature, primarily defined by the constant interaction with a heavy, complex external object—the bicycle.

This human-bicycle interface creates three distinct challenges that separate BMX from seemingly similar sports like gymnastics or skateboarding:

1. A Dual-Mass Propulsion System: Unlike gymnasts who propel only their body mass from a stable surface, BMX athletes must generate explosive power sufficient to lift a combined system of rider and bicycle. This transforms power generation from a pure lower-body action into a whole-body task, requiring significant force contribution from the upper body and core to pull and maneuver the bike frame, a fact supported by electromyography studies showing high activation in upper limb and core musculature during maneuvers (Voronov et al., 2020).
2. The Athlete as the Suspension System: BMX bicycles lack any form of shock absorption. Consequently, the athlete’s musculoskeletal system must function as the sole shock absorber during high-impact landings, which can generate ground reaction forces exceeding eight times body weight in analogous sports like snowboarding (Noonan, 2018). This imposes extreme eccentric strength demands and unique joint loading patterns not seen in sports with purpose-built sprung floors or mats.
3. Disassociated Rotational Dynamics: While many sports involve rotations, BMX Freestyle requires athletes to perform complex maneuvers (e.g., tailwhips, barspins) where the bike and rider can rotate independently. This demands a level of coordination to manage two separate moments of inertia. Successfully controlling these disassociated rotations requires immense core stability to manipulate the body’s moment of inertia independently of the bicycle’s, a critical component of advanced aerial skill execution (Sweeney, 2020; Voronov et al., 2020).

These unique demands directly point to critical knowledge gaps: the precise contribution of the upper body to power generation remains unquantified; the biomechanics of impact absorption and its relation to specific injury patterns are poorly understood; and the neuromuscular sequencing for complex, disassociated tricks has not been mapped. Therefore, understanding the BMX Freestyle athlete requires moving beyond generic athletic models and focusing on this unique human-bicycle dynamic.

In BMX Freestyle, the interplay between physiological capabilities and technical skills necessitates integrated training methodologies. Athletes must not only maximize anaerobic capacity and neuromuscular efficiency through targeted strength and conditioning regimens but also hone technical proficiency via on-bike practice and drill-specific training. For instance, training programs that combine plyometric exercises (to enhance lower-limb explosive power) with balance training (to improve core stability and dynamic equilibrium) may be particularly advantageous for BMX athletes (Cowell et al., 2012; Gross and Gross, 2019). Furthermore, the integration of cognitive training strategies aimed at improving reaction time and decision-making speed can further augment an athlete’s ability to execute complex tricks under competitive conditions (Semaeva et al., 2023).

### Skill acquisition and coaching for the BMX freestyle athlete

Beyond the physiological profile, a holistic understanding of athlete development requires appreciating the unique context in which skills are acquired. As the literature on action sports suggests, learning environments are often informal and athlete-driven, contrasting with the highly structured models of traditional sports (Ellmer et al., 2020). This is vividly illustrated in the Australian BMX context, where athletes have historically relied on self-coaching and peer-coaching to progress. A critical tool in both paradigms is the use of video analysis; athletes are frequently observed filming their own attempts to reflect on technique, or recording their peers to engage in collaborative problem-solving and provide immediate, corrective feedback (Ellmer, 2025). This informal, technology-integrated, and peer-led learning culture means that even in high-performance settings, the formal coach must often act as a facilitator who fosters athlete autonomy, rather than a top-down director (Ellmer, 2025). Therefore, for the physiological insights and training recommendations of this review to be successfully implemented, they must be delivered within a coaching framework that respects this athlete-centered learning process.

### Practical applications and implications

The synthesis of the physiological and physical characteristics of BMX Freestyle athletes provides a clear, evidence-based framework for practical applications that extend beyond theoretical understanding and can directly inform athletic development.

#### Optimization of training protocols

The unique demands of the sport necessitate a departure from generic conditioning towards highly specific training programs. Given the importance of explosive power, training should integrate plyometrics and weighted jumps, which have been consistently shown to improve vertical jump performance and power output (Ramirez-Campillo et al., 2020). To address the challenge of the “athlete as suspension,” a focus on eccentric strength training is crucial, as it is highly effective at increasing eccentric force production and inducing muscular adaptations associated with injury prevention (Douglas et al., 2017). Furthermore, the paramount importance of core stability for executing complex rotations dictates that training regimens include dynamic and anti-rotation exercises, as it is fundamental to controlling body segments and facilitating force transfer (Huxel Bliven and Anderson, 2013). Indeed, core training that integrates ‘body-centering’ (the conscious regulation of intra-abdominal pressure for postural stability) has been shown to more effectively enhance balance, trunk control, and lower limb explosive power than core training performed in isolation (Fogliata et al., 2025).

#### Data-driven talent identification

The identified athletic profile can inform more effective and objective talent identification models. Rather than relying solely on subjective observation, federations and clubs could implement a battery of quantitative tests, a practice supported by systematic reviews as a cornerstone of modern talent identification programs (Johnston et al., 2018). Key performance indicators could include vertical jump height as a proxy for explosive power, dynamic balance tests like the Star Excursion Balance Test, and assessments of relative upper-body strength. This data-driven approach, as successfully applied in the analogous sport of skateboarding (Ab Rasid et al., 2024), can help objectively identify young athletes who possess the foundational physical attributes for future success in BMX Freestyle.

#### Targeted injury prevention strategies

A clear understanding of the sport’s unique demands allows for the development of targeted injury prevention strategies. The high impact forces and rotational stresses highlight the need for neuromuscular training programs that focus on strengthening the stabilizing muscles around the ankles, knees, and trunk, which has been proven effective in reducing lower limb injuries in sports (Hübscher et al., 2010; Kamper and Moseley, 2011). This is particularly critical for the upper extremities, as a retrospective analysis of the Tokyo 2020 Olympics found that 66.7% of injuries sustained by BMX Freestyle athletes occurred in the upper limbs, underscoring the unique demands of bike control and impact absorption (Tanaka et al., 2024). Furthermore, given that a lack of core strength or grip endurance can lead to catastrophic failures during trick execution, targeted core and grip endurance training should be considered a primary pillar of any injury prevention program, moving beyond general pre-habilitation to address the specific failure points in BMX Freestyle.

### Limitations and future research directions

While this review establishes a foundational profile, it also illuminates several critical knowledge gaps that hinder the sport’s scientific advancement. Of particular concern is the profound scarcity of comprehensive data pertaining to female BMX Freestyle athletes, whose peak competitive age also appears to occur earlier. As female participation in the sport continues to escalate (Fouillot et al., 2025), such data are indispensable for the formulation of evidence-based training guidelines, talent identification protocols, and robust injury prevention strategies. Detailed kinematic and kinetic analyses of diverse tricks are warranted to elucidate joint loading patterns, force magnitudes, and optimal technical execution models. Concurrently, investigations into landing strategies and impact absorption mechanisms are equally imperative. Such research would offer profound insights into injury etiology, given the sport’s reportedly high injury rates, and provide a scientific foundation for refining technical instruction aimed at enhancing performance and safeguarding athletes. While existing EMG studies (Voronov et al., 2020) represent a positive initial step in understanding muscle activation, comprehensive biomechanical analyses remain to be undertaken. Furthermore, there is a pressing need to develop and validate sport-specific assessment protocols for BMX Freestyle to evaluate attributes such as agility, dynamic balance, coordination, and reactive strength. Certain generic tests currently employed, such as the static balance assessments previously discussed (Albaladejo-García et al., 2023), may not adequately capture the unique demands of this dynamic sport. Methodologies with greater ecological validity would yield more meaningful data for athlete assessment and the evaluation of training efficacy.

Although this review primarily addresses physiological characteristics, the cognitive demands and competitive pressures inherent in the sport, as alluded to previously, suggest that psychological factors are of comparable importance. The psychological skill set of elite athletes may indeed be a critical differentiating factor in their performance outcomes. While reviews such as that by Rockliff et al. (2024) have examined injury risks in general BMX cycling, and initial Olympic data is now available (Tanaka et al., 2024), more detailed and specific injury surveillance data for BMX Freestyle are required to delineate injury types, etiological mechanisms, and severity levels. Concurrently, evaluations of the efficacy of protective equipment, such as the helmet mandates stipulated by UCI regulations, should be intensified. Given the sport’s high reported injury rates, targeted preventative research is imperative and warrants urgent attention. This research gap is formally recognized within the UCI’s 2030 Agenda and has been highlighted by a recent systematic review protocol, which notes a profound lack of epidemiological research in lesser-known disciplines like BMX Freestyle (Fallon and Heron, 2024).

Furthermore, while BMX Freestyle possesses distinct characteristics, methodological approaches or general principles pertaining to aspects such as the biomechanics of rotational movements, impact absorption, and agility training might be judiciously adapted from research in analogous ‘extreme’ or ‘skill-based’ sports (e.g., skateboarding, freestyle skiing, gymnastics). However, any such extrapolation necessitates rigorous sport-specific validation, given the unique human-bicycle interface that defines BMX Freestyle’s particularities.

### Implications of an evolving sport

Finally, BMX Freestyle is a discipline characterized by the rapid evolution of trick difficulty. It has been observed that “change is constant,” and athletes are “all in pursuit of the perfect run.” The emergence of maneuvers such as ‘Cork 720s with triple barspins’ and ‘360 double tailwhips to opposite tailwhips,’ sometimes described as “tricks seemingly out of a video game,” signifies a continuous escalation in technical complexity. This relentless pursuit of novelty and difficulty translates into synchronously increasing demands on athletes’ physiological, biomechanical, and neuromuscular control capabilities. Consequently, earlier research may not fully encapsulate the challenges currently faced by elite competitors. This, in turn, imposes new imperatives for future research and talent development programs, which must prepare athletes for an ever-advancing ceiling of athletic performance.

Ultimately, success in BMX Freestyle hinges on a delicate equilibrium between innate physiological capacities and meticulously honed technical skills-an equilibrium best achieved through comprehensive training programs that address both the metabolic and biomechanical facets of this dynamic sport (Camilleri et al., 2024; Semaeva et al., 2023). Continued investigation into the specific characteristics of BMX Freestyle will not only deepen our understanding of the physiological and physical attributes requisite for elite performance but also inform the development of coaching and conditioning frameworks capable of nurturing the next-generation of talent (Brasil et al., 2020). As BMX Freestyle continues its global expansion and solidifies its Olympic status, conducting in-depth scientific research to fill existing knowledge lacunae is of paramount importance. Such endeavors are crucial for optimizing training methodologies, refining scientific talent identification processes, implementing effective injury prevention strategies, and ultimately, advancing the health and competitive excellence of athletes in this unique sporting discipline.

## Conclusion

In summary, a physique conducive to a high power-to-weight ratio, pronounced agility, and the capacity to withstand substantial impact forces is advantageous for BMX Freestyle. Physiologically, athletes typically exhibit high anaerobic power outputs, essential for supporting the execution of short-duration, explosive maneuvers. A moderate level of maximal oxygen uptake (VO_2_max) is likely utilized primarily for recovery during training sessions and for sustaining performance across multiple runs in competition. Athletes generally possess robust lower-limb explosive power and significant upper-body strength, which are critical for bicycle manipulation and the absorption of landing impacts. Maximal grip strength and endurance are also paramount, complemented by highly developed core strength necessary for executing rotational movements and maintaining postural stability. Further key attributes include highly developed, task-specific dynamic balance and coordination, precisely modulated muscle activation patterns tailored to trick complexity and type, and adequate flexibility. Collectively, these characteristics enable athletes to execute high-difficulty, large-amplitude rotational tricks, maintain precise vehicular control within dynamic environments, and endure the rigors of training and competition. Deficiencies in any of these areas can culminate in failed attempts, reduced scores, or an elevated risk of injury. As the sport continues to evolve, future research should not only aim to fill the descriptive gaps identified in this review but also focus explicitly on applied science outcomes. This includes designing and validating targeted training interventions to enhance lower-limb explosive power and eccentric strength, and developing evidence-based injury prevention programs that address the unique landing mechanics of the sport. Longitudinal studies tracking athletes through such optimized protocols are crucial to confirm their efficacy and to ultimately advance the health, safety, and competitive excellence of athletes in this unique sporting discipline.
